# Heat Shock Protein 90 in Plants: Molecular Mechanisms and Roles in Stress Responses

**DOI:** 10.3390/ijms131215706

**Published:** 2012-11-23

**Authors:** Zhao-Shi Xu, Zhi-Yong Li, Yang Chen, Ming Chen, Lian-Cheng Li, You-Zhi Ma

**Affiliations:** Institute of Crop Science, Chinese Academy of Agricultural Sciences (CAAS)/National Key Facility for Crop Gene Resources and Genetic Improvement, Key Laboratory of Biology and Genetic Improvement of Triticeae Crops, Ministry of Agriculture, Beijing 100081, China; E-Mails: xuzhaoshi@yahoo.com.cn (Z.-S.X.); wangwangsd2010@yahoo.com.cn (Z.-Y.L.); chenyang712@126.com (Y.C.); chenming@mail.caas.net.cn (M.C.); lilch@mail.caas.net.cn (L.-C.L.)

**Keywords:** plant Hsp90, interaction mechanism, disease resistance, abiotic stress tolerance, phylogenetic relationship

## Abstract

The heat shock protein 90 (Hsp90) family mediates stress signal transduction, and plays important roles in the control of normal growth of human cells and in promoting development of tumor cells. Hsp90s have become a currently important subject in cellular immunity, signal transduction, and anti-cancer research. Studies on the physiological functions of Hsp90s began much later in plants than in animals and fungi. Significant progress has been made in understanding complex mechanisms of HSP90s in plants, including ATPase-coupled conformational changes and interactions with cochaperone proteins. A wide range of signaling proteins interact with HSP90s. Recent studies revealed that plant Hsp90s are important in plant development, environmental stress response, and disease and pest resistance. In this study, the plant HSP90 family was classified into three clusters on the basis of phylogenetic relationships, gene structure, and biological functions. We discuss the molecular functions of Hsp90s, and systematically review recent progress of Hsp90 research in plants.

## 1. Introduction

Heat shock proteins (Hsps) are widely distributed in fungi, animals, and plants. Hsp transcripts significantly upregulate under high-temperature stress, and play an important role in heat stress response in living cells [1]. Hsps are mainly located in the cytoplasm under normal physiological conditions, but rapidly transfer to the nucleus under stress conditions. In accordance with molecular weight, Hsps are divided into several families, such as Hsp110, Hsp90, Hsp70/Hsp80, Hsp60, and small molecular Hsp (smHsp). The smHsp family were initially proposed to serve as molecular chaperones [2,3]. Now it has been demonstrated that Hsp90, Hsp70, and Hsp60 also function as molecular chaperones. Since the role of *Arabidopsis* Hsp90 in stress responses is known from the early studies, recent studies have showed that Hsp90 might play an important role in biological stress responses.

Hsp90s, highly conserved in molecular evolution, are involved in regulating and maintaining conformation of a variety of proteins, and in assisting normal cell survival under stresses [4]. In fungi and animals, Hsp90s mediate extensive stress signal transduction, including a role in folding of steroid hormone receptors, protein kinases, and transcription factors, as well as activation of the substrate to initiate stress signal transduction [5–8]. Recent studies showed that Hsp90s play an important role in controlling normal growth of human cell and in promoting tumor cell development [8,9]. Many oncoproteins are targets of Hsp90s, and Hsp90 inhibition can result in multipathway anti-tumor effects [10,11]. Inhibition of Hsp90 activity contributes to degradation of oncoproteins, helping in cancer treatment [5,8,12,13]. Cellular functions of the molecular chaperone activities of Hsp90s have been intensively studied in fungi and mammalian model systems. In contrast, studies on the physiological functions of Hsp90s in plants are more recent. A number of *HSP90* genes have been identified from many plants, and they were strongly induced by changes in temperature, salinity, and heavy metals [14–18]. Molecular mechanisms of plant Hsp90s in response to stress, their roles as molecular chaperones, and their functions in enhancing plant resistance are not clear. It was recently shown that Hsp90s play an important role in plant development, stress response, and disease resistance [19–21].

Hsp90s directly influence canalization, assimilation, and rapid evolutionary alterations of phenotype through concealment and exposure of cryptic genetic variation [22]. Therefore, in-depth investigation of plant Hsp90 functions may help in understanding stress signal transduction, discovering pivotal stress-related genes, and improving the crop resistance. This paper reviews recent research on the molecular mechanisms of plant Hsp90s in order to promote their study, especially in regard to their manipulation in achieving biotic and abiotic stress resistance in crop plants.

## 2. Structural and Functional Analyses of Hsp90

Hsp90s are abundant (*i.e*., ~1% of total proteins) in prokaryotic and eukaryotic cells. They are essential for viability in eukaryotes. Hsp90 increases to as much as 4%–6% of total protein under heat stress conditions [6,23]. They usually maintain a homodimer form that greatly increases in an emergency. They are mainly present in cytoplasm, and only rarely appear in the endoplasmic reticulum, mitochondria, and chloroplasts [24,25]. Hsp90s in the cytoplasm of animals is divided into two subtypes: Hsp90α and Hsp90β. Hsp90αs are sensitive to heat stimulation, whereas Hsp90βs respond to mitotic induction. Hsp90αs mainly contribute to protein transportation, protein folding, and maintenance of normal conformation preventing aggregation with ATP. Hsp90β is involved in the utilization of steroid hormones [26]. Hsp90s play different roles in prokaryotes and eukaryotes and are not necessary for the growth of prokaryotes [27,28].

**Figure 1:**
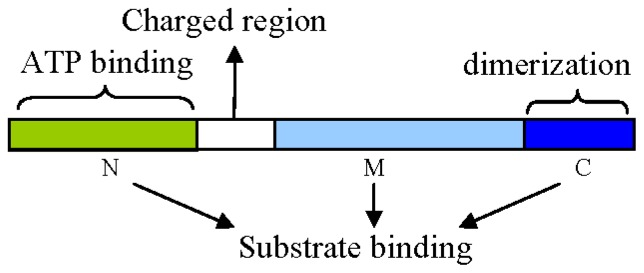
Structural model of Hsp90. Hsp90 consist of three domains: an *N*-terminal ATP-binding domain (N); a middle domain (M); and a *C*-terminal dimerization domain (C) with the pentapeptide MEEVD sequence. A charged region exists between the N and M domains. All three domains are reported to interact with different substrates.

Hsp90s serve as molecular chaperones in dimers regulated by ATP. Almost all of the Hsp90 family members are conserved, containing three domains (Figure 1): viz. an *N* terminal conserved ATP-binding domain, a middle domain (M), and a *C*-terminal dimerization domain [23,29–31].

The ATP-binding domain of the Hsp90 *N*-terminal is the ATP/ADP binding site with intrinsic ATPase activity. Conformational changes in Hsp90s occur in the presence of ATP and then contribute to signal transduction, protein folding, and morphological evolution. ATP binding and hydrolysis cause conformational transitions of Hsp90 between an open conformation with the *N*-domains separated and a closed conformation with the *N*-domains associated [32,33]. Mutation analysis showed that ATPase activity was necessary for the biological functions of Hsp90 [29,34]. Some natural substances, such as geldanamycin, closely combine with this position to interfere with Hsp90 functions, and then promote rapid degradation of protein kinase [35].

The middle domain of Hsp90 plays a key role in binding to substrate. Hsp90 and DNA topoisomerase II gyrase B (GyrB) share a similar structure, indicating the middle domain of Hsp90 as a primary substrate binding site. The middle domain contains a catalytic ring which can sense the presence of ATP-γ phosphate and combine with it. The function of Hsp90 depends not only on the *N*-terminal domain, but also on the participation of the middle domain [36,37].

The *C*-terminal domain of Hsp90 is necessary for dimerization, and also functions as the binding site of calmodulin and other substrates. In eukaryotic cytoplasm, the *C*-terminal domain of Hsp90 contains a conserved five peptide domain (MEEVD) that is essential for the interaction between Hsp90 and proteins containing a tetratricopeptide repeat (TPR) domain. HSP70 also interacts with the TPR domain by an eight amino acid domain (GPTIEEVD) in a similar way to Hsp90 [5,29].

**Figure 2:**
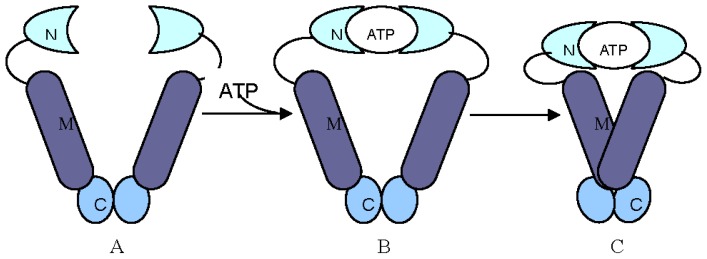
The Hsp90 molecular clamp driven by ATP [8]. In the absence of ATP (**A**), Hsp90 is dimerized at its C-terminus in the “open” conformation. Upon ATP binding, the *N*-terminal domains undergo conformational changes that result in closing a “lid” over the bound nucleotide, and formation of a second dimerization interface between the amino-termini (**B**). Continued rearrangements of the closed conformation allow interaction of the N-terminal and middle domains resulting in the “closed and twisted” conformation which is able to hydrolyze ATP (**C**).

There is a charge zone consisting of about 50 amino acid residues between the *N*-terminal domain and the middle domain of Hsp90 in eukaryotes [38]. This area is not necessary for Hsp90, and mainly assists in covalent linkages, coordinating the *N*-terminal domain and the middle domain to maintain the conformation of Hsp90 binding with ATP. In fact, all three domains (*N*-terminal, middle region and *C*-terminal) of Hsp90 were reported to be involved in binding with substrate proteins [29]. A general theory of interaction between Hsp90s and substrate proteins was formulated [8]: Hsp90s cannot form dimers without combining with ATP, and capture substrate proteins by two separate *N*-terminals. When the *N*-terminal of Hsp90 is combined with ATP, its conformation is changed to a ring formation to wrap the substrate protein. Hsp90 has to bind with the substrate to maintain a so-called “non-active state” (Figure 2).

## 3. Interactions of Proteins with Hsp90s and Their Interaction Mechanism

**Figure 3:**
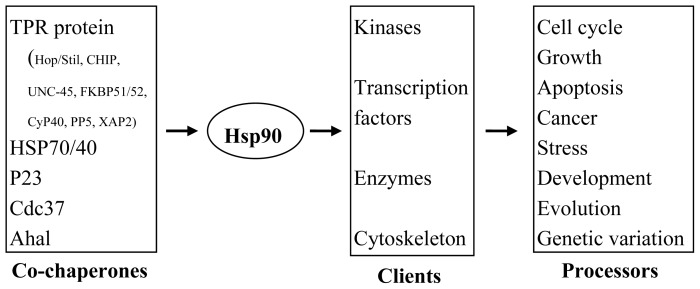
Functions of Hsp90 and co-chaperones identified by interaction with substrates in different developmental phases in cells.

Hsp90 participates in signal transduction indirectly by interaction with other proteins. Therefore, the loss of Hsp90 function may cause a variety of physiological defects in cells. More than 600 proteins interact with Hsp90 in yeast two-hybrid tests, tandem affinity chromatography, and genetic analyses in yeast [39]. Proteins interacting with Hsp90, mainly group into three types, include auxiliary proteins (co-chaperones), regulatory factors (regulators), and substrate proteins (substrates) (Table 1). Auxiliary proteins aid Hsp90 by binding with substrate proteins to maintain their stability. Many studies show that Hsp90 is regulated by phosphorylation, but the mechanism of regulation is still not clear. Eukaryotic Hsp90 proteins require accessory proteins and regulatory factors to regulate the interaction between themselves and substrate proteins, which together confer the diverse physiological activities of Hsp90 (Figure 3).

### 3.1. Accessory Proteins

Accessory proteins play a key role in regulating the ATP enzymatic activity of Hsp90s in cytoplasm and in mediating interactions between Hsp90s and substrates [8,40]. Most of the accessory proteins contain a TPR domain, and mainly depend on this domain to combine with the conservative *C*-terminal pentapeptide MEEVD of Hsp90. Each Hsp90 contains only one specific TPR-binding site. The TPR domain-containing proteins that interact with Hsp90 include HOP protein (corresponding to yeast Sti1), pro-free prime FKBP51 and FKBP52, cyclophilin Cyp40 (corresponding to yeast Cpr6 and Cpr7), protein phosphatase 5, yeast Cns1, Drosophila Dpit47, E3 ubiquitin ligase CHIP, and myosin-binding protein UNC-45. Although there are many TPR domain-containing proteins in plants, there are few reports besides ROF1 about their interaction with Hsp90 [41].

P23, a small acidic protein with chaperone activity, combines with the middle section of the *N*-terminal domain of Hsp90. It specifically identifies the binding state of Hsp90-ATP and plays a special role in the regulation of the Hsp90 ATPase cycle to promote the assembly and decomposition of the steroid receptor transcription complex [42]. Recent studies revealed that the activity of PIDD (p53-induced protein with a cell death domain) was regulated by a p23-Hsp90 complex [43] and PIDD mediated the activation of DNA repair by NF-kappaB [44]. The *C*-terminal of Cdc37 combines with the flanking region of Hsp90 at the *N*-terminal and may be the auxiliary protein of one specific substrate of Hsp90. Cdc37 regulates Hsp90 in specifically combining with protein kinase [45]. Recent research showed that Hsp90 allied with cyclophilin A to promote selective transportation of substrate, but the exact mechanism remains unclear [46]. These observations reveal that accessory proteins competitively binding to Hsp90 or substrate proteins generate different physiological functions.

### 3.2. Regulators

Hsp90s serve as chaperones depending on phosphorylation and stimulate heme to regulate HRI under phosphorylation by casein kinase II or secondary phosphorylation [47]. Phosphorylation of Hsp90s induces the release of substrate proteins, such as pp60v-src and reovirus protein σ1 [48]. Sch9 kinase, which inhibits the function of Hsp90 in yeast [49], cooperated to regulate PKA activity [50]. Treatment with the Hsp90 inhibitor radicicol upregulated 114 genes in yeast, and among them 26 contained Msn2/Msn4 binding elements [51]. More recently, a report demonstrated that an Hsp90 mutation selectively disrupted cAMP/PKA signaling in *Saccharomyces cerevisiae*, indicating that a specific interaction of Hsp90 and Sgt1 with Cyr1 plays a key role in regulating gene expression [52]. However, the phosphorylation sites of Hsp90s and the molecular mechanism of most kinases have yet to be elucidated.

### 3.3. Substrate Proteins

Hsp90s affect the folding and activation of a wide variety of substrate proteins, most of which are kinases and transcription factors involved in signal transduction and regulatory processes [5,53,54]. Correspondingly, the stabilized conformation of these substrates require HSP90 [55]. It was reported that PKB/AKTs combine with the middle domain of Hsp90s to regulate their kinase activity by proteasome-mediated degradation [56]. Src family members require the participation of Hsp90 to refold them during transportation through membranes. Hsp90 inhibited PKR kinase activated by dsRNA to maintain a mature form of PKR [57]. Recent, research showed that Hsp90-p23 interacted with hTERT (telomerase reverse transcriptase) to regulate the activity of telomerase in maintaining its normal behavior [58]. Additionally, the conformational plasticity of Hsp90s may allow them to adapt to structurally diverse substrates, thereby catalyzing further structural changes that lead to ATP hydrolysis [59]. Folding of most proteins in yeast does not require involvement of Hsp90, but Hsp90 can promote the damage repair of proteins under stress. However, fundamental distinction between proteins dependent and independent of Hsp90 is in need of further research.

Hsp90s in animals are extensively involved in signal transduction, protein synthesis and folding, and DNA repair, functions that highlight their growing significance [4,5,8,43]. Now that Hsp90 is a target of the antitumor drug geldanamycin [35], increasing attention is focusing on the group. However, the biological functions of Hsp90s in plants are less well identified. At present, these are supposed to mainly involve assisted protein folding, and protein complex assembly and degradation, as well as activation of substrates or enhancing the biological activity of substrate proteins [60,61].

## 4. The Hsp90 Family in Model Plants

Although Hsp90s have been identified in various plant species, the evolutionary relationships of Hsp90 among them are uncertain. Three representative dicotyledons (*Arabidopsis*, soybean and grapevine) and two monocotyledons (rice and maize) were selected for study. Four databases were searched to identify Hsp90 genes: viz., TAIR (the *Arabidopsis* Information Resource) [62], Rice Genome Annotation Project Database [63], Phytozome [64], and Maize Genome Annotation Project Database [65]. When there was more than one allele, the longest was chosen as representative. Seven Hsp90 genes were identified in *Arabidopsis* (Table 2) [66], and 15, nine, and 12 putative Hsp90 genes were identified in soybean (Table 3), rice (Table 4), and maize (Table 5), respectively. Hsp90 genes in grapevine were analyzed by Banilas *et al.*[18]. Predictions of the subcellular locations of plant Hsp90 proteins were made using PSORT [67].

**Figure 4:**
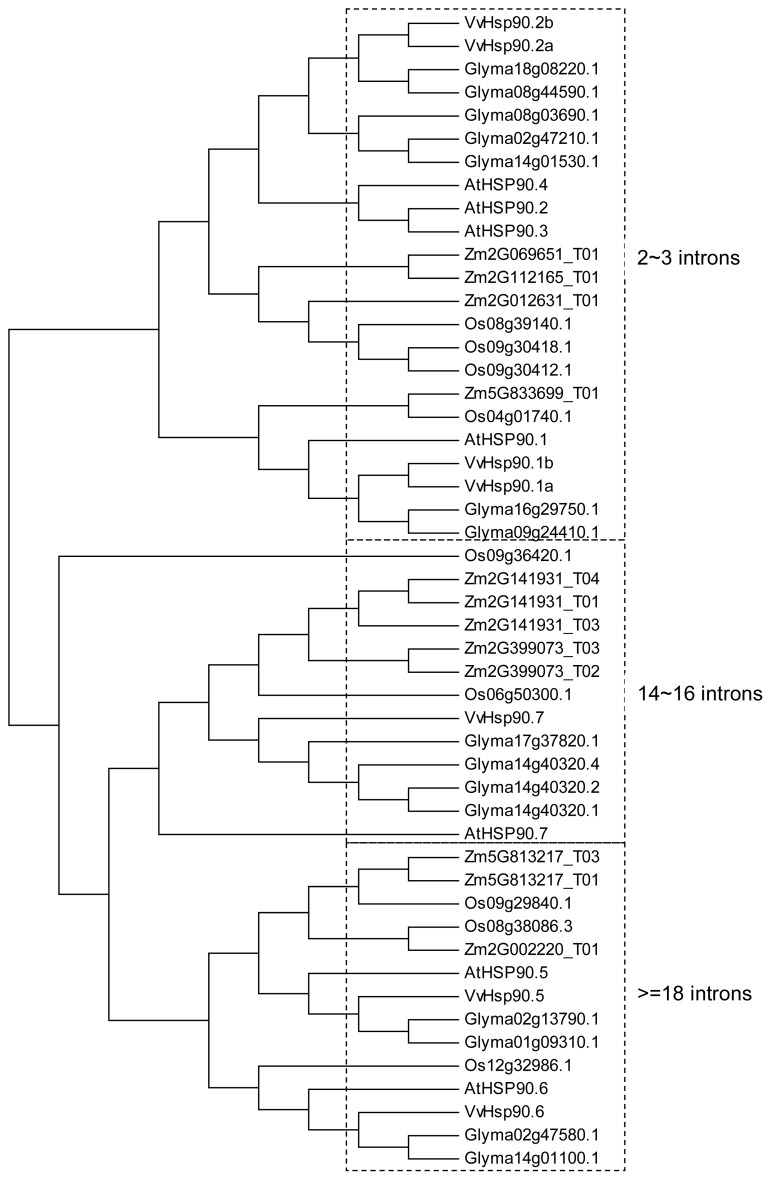
Phylogenetic tree of Hsp90 genes from model plants. Alignments of protein sequences were performed using the CLUSTALW program [68] with default parameters. The rectangular phylogram of the Hsp90 protein sequences was generated using MEGA 4.1 [69]. Abbreviations: AT, *Arabidopsis thaliana*; Os, *Oryza sativa*; Glyma, *Glycine max*; Zm, *Zea mays*.

We performed multiple alignment analyses using full-length amino acid sequences. The alignments indicated that Hsp90s could be placed in 3 clusters (I to III) and 10 sub-clusters according to their phylogenetic relationships, gene structures, conserved motifs, and biological functions (Figure 4). Gene structures of clusters I, II, and III were characterized by having more than 18, 14 to 16, and 2 to 3 introns, respectively, suggesting that intron numbers were established in each cluster before divergence of dicotyledons and monocotyledons.

Homologous genes from the dicotyledons shared high homology, and likewise the monocotyledons, indicating that the Hsp90 genes evolved further in species-specific ways, although gene structure organization and expression characteristics of Hsp90s are similar in different plants. However, the relationships of homologs are less clear. For example, rice Os12g32986.1 was homologous to *Arabidopsis* At3G07770, soybean Glyma02g47580.1 and Glyma14g01100.1, but Os12g32986.1 protein was localized in the vacuole whereas its counterparts from *Arabidopsis* and soybean (At3G07770, Glyma02g47580.1, and Glyma14g01100.1) accumulated in the mitochondrial matrix space and nucleus, respectively.

Over the past decade, investigation of *Arabidopsis* Hsp90s has attracted the most attention. AtHSP90-2, −3, and −4 have high similarity with homology of about 96%, implying functional redundancy. AtHSP90-1, −2, −3, and −4 contain the specific target signal MEEVD, essential for sub-cellular location in the cytoplasm, at the *C*-terminal. AtHsp90-5, −6, and −7 were predicted to be located within the plastidial, mitochondrial, and endoplasmic reticulum compartments, respectively, based on their *N*-terminal signal peptides [20,55].

Loss of Hsp90 function in plants leads to deformities and affects physiological characteristics [4,5]. Inhibition in *Arabidopsis* resulted in an abnormal plant phenotype, including an epinastic cotyledon, disc or radial symmetry of cotyledons, and abnormal growth of root hairs [70]. Hsp90-RNAi lines had increased accumulation of purple pigment in the cotyledons with later development of true, but narrower leaves. The developing plants lacked apical dominance, and produced multiple primary inflorescences with abnormal stems and increased numbers of rosette leaves. They were also later flowering [71].

Hsp90 is also involved in embryo development and seed germination. A dramatic increase in *AtHsp90*-*1* transcript was observed in embryos during their development, whereas *AtHsp90*-*3* increased slowly during pod elongation and decreased slightly in nearly mature embryos. It was concluded that Hsp90 participated in the process of seed embryo formation and seed germination [72]. Hsp90 also affected elongation of the hypocotyl [73]. Also, it was recently shown that constitutive overexpression of *AtHsp90*-*3* resulted in lower germination rate and shorter root length in *Arabidopsis*[74]. Thus, Hsp90 appears to be extensively involved in plant growth and development.

## 5. Hsp90 Is Involved in Plant Stress Resistance

Hsp90 are usually maintained in the dipolymer form and are sharply induced under extreme conditions. Many Hsps, including Hsp90, are induced by heat shock stress and are involved in regulation of heat shock response. There are several heat shock elements (HSEs) bound with heat shock factors (HSFs) in the promoters of Hsps. In normal conditions, the HSF monomer is combined with Hsp90 dimers; but under stress conditions, the HSF monomer converts to a trimer and closely integrates with HSE to initiate transcription of Hsp genes [21,75]. HSP is induced not only by heat shock, but also by many other stresses.

It seems that most of the substrates of Hsp90s are kinases and transcription factors that function pivotally in signal transduction to activate or suppress defense gene expression, as well as in the regulation of interaction between different signaling pathways [76]. Clearly, Hsp90s mediate both abiotic and biotic stress resistances in plants.

### 5.1. Hsp90s Are Involved in Disease and Pest Resistances

In recent years, it has become apparent that Hsp90s mediate signal transduction pathways of disease resistance in plants. Plant immunity is initiated by resistance (R) proteins that recognize pathogen effector proteins. The interaction of Hsp90 and SGT1 (suppressor of G2 allele kinetochore protein) and RAR1 (required for *Mla12* resistance) confers stability to R protein contributing to the recognition of pathogen eggectors. SGT1 interacts with Hsp90 through its CS domain and TPR domain binding with the ATPase site and *C*-terminal of Hsp90, respectively, and SGT1 binds with the R protein at the LRR (leucine rich repeat) domain, while RAR1 recognizes the ATP binding region of Hsp90 [77]. Hsp90 complexes in *Arabidopsis* directly regulate the resistance protein activity and play a key role in assisting in R-mediated disease resistance [78]. Disease resistance mediated by RPM1 was weakened in AtHsp90-2 mutant plants [79].

Hsp90 is required for regulation of resistance to *Pseudomonas cloves* conferred by the tomato *Pto* gene [80]. The interaction of Hsp90 with RAR1 and TIR-NB-LRR in tobacco improved mosaic virus (TMV) resistance in tobacco [81]. It was proved through BSMV-VIGS technology that the SGTl, RARl, and Hsp90 genes are necessary for *Mla13*-mediated powdery mildew resistance in barley and *Lr21*-mediated leaf rust resistance in wheat [82–84].

Recent studies revealed that Hsp90 are also involved in signal transduction pathways of plant resistance to insects. Tomato Mi-1 protein containing a nucleotide binding site (nucleotide-binding site, NBS) and LRR motifs mediate resistance to root-knot nematodes, potato aphid and whitefly. Knockouts of SGTl and Hsp90 weakened the Mi-1-mediated resistance to root-knot nematode and potato aphid, whereas RARl was not necessary [85]. The mechanisms involved in these responses require further detailed study.

### 5.2. Hsp90s Mediate Abiotic Stress Resistance

Hsp90s mediate plant abiotic stress signal pathways [86], but again the mechanisms are unclear. A Hsp90-specific inhibitor in the cytoplasm induced the accumulation of heat-induced gene products and improved heat tolerance in *Arabidopsis*[87,88]. Under normal conditions, Hsp90.2 in the cytoplasm negatively regulates transcription of heat-induced genes by suppression of HSF. Hsp90.2 is instantaneously inactivated under heat shock, and HSF is activated to induce expression of downstream genes containing HSF elements [87]. It was subsequently confirmed that overexpression of Hsp90.2 suppresses HsfA2 transcription, and that HsfA2 is induced under inhibition of Hsp90.2 in order to adapt to oxidative stress [88]. Similarly, an inhibitor of Hsp90 produced by a rhizosphere fungus repressed the growth and development of plants, but enhanced the heat resistance of *Arabidopsis*[89]. These studies show that Hsp90s play important roles in adaptation of plants to stress environments.

Although Hsp90s are involved in abiotic stress responses in plants, little research on improving abiotic stress resistance has been reported. The expression of *UpHSP90* was strongly and positively regulated by diurnal and temperature changes, and slightly influenced by long-term treatment with heavy metal stress in sterile *Ulva pertusa*[90]. Overexpression of *rHsp90* from rice significantly increased salt stress in tobacco [91]. Yeast Hsp90 was involved in yeast cell wall permeability and stress response through the HOG and MAPK pathways [92]. The MAPK signal pathway occurs in plants. Recent study showed that the MAPK cascade functions down-stream of Hsp90 and transduces the cell death signal to mitochondria for *N* gene-dependent cell death [93].

The way by which auxiliary proteins regulate the ATPase activity of Hsp90 in cytoplasm to assist Hsp90 to interact with the substrates is similar in animals and plants. *Arabidopsis* FKBP (FK506 binding protein) family members ROF1 (FKBP62) interact with Hsp90.1 through the TPR domain [41]. Under normal conditions the ROF1-Hsp90.1 complex targets the cytoplasm. When stimulated by heat, HsfA2 combines with the complex to form a ROF1-Hsp90.1-HsfA2 complex that migrates to the nucleus and regulates sHsp transcription to enhance heat tolerance [94]. Silencing of ROF1 or ROF2 (FKBP65, ROF1 homologous protein) increases the heat tolerance of plants, but ROF2 nuclear localization is independent of Hsp90.1 or HsfA2 which negatively regulate activation of HsfA2 and inhibit transcription of sHsp expression [95].

## 6. Prospects

In animals and fungi, Hsp90s generally mediate stress signal transduction, folding of transcription factors and protein kinases. High expression of human Hsp90 is closely related to tumor development and may enhance the anti-apoptosis capacity of tumor cells, possibly offering a new therapy for treatment of cancer. The interactions of numerous proteins with Hsp90s were discovered only recently. These interactions provide opportunities for in-depth study of the functions and mechanisms of Hsp90s. Similarly, plant Hsp90s are involved in a variety of biological processes related to growth and development, as well as various responses to environmental stress. In *Arabidopsis*, overexpression of *AtHsp90.2*, *AtHsp90.5* or *AtHsp90.7* reduced plant tolerance to drought stress [96]. When grapevine was suffered from drought combined with heat stress, the up-regulation of *VvHsp901a* was delayed [18]. These results provide insights into the roles of Hsp90 machinery in plant development and response to adversity stress, and promote further research to better understand the molecular mechanisms of plant response to environmental stress.

Knowledge of Hsp90 in plants is much less advanced and there are fewer reports about the physiological functions of Hsp90 chaperone complexes. Although the results of recent studies revealing that Hsp90s could improve plant disease resistance and buffer genetic variation have gained attracted the interest of plant biologists, the potential roles of plant Hsp90s are still not clear; especially the characteristics of all plant Hsp90s and the Hsp90-mediated signal pathways. It is of theoretical and practical significance that in-depth investigation of the molecular relationship among Hsp90s, environmental stresses, and resistance in plants may contribute to molecular breeding for stress resistance.

## Figures and Tables

**Table 1:** Proteins interacting with Hsp90s.

Chaperones and relatives:	Transcription factors:
- Cdc37 (= p50) - Cdc37 relative Harc - Hsp70 - Human DnaJ homolog Hsj1b - p23/Sba1 - Proteins with tetratricopeptide motifs: Hop/Sti1, FKBP51, FKBP52 (+ high MW plant homologs), cyclophilin-40/Cpr6 and Cpr7, protein phosphatase 5, Tom70, Ah receptor interacting protein AIP, Cns1 (and its Drosophila relative Dpit47), CHIP, UNC-45/She4 - Sse1	- 12 (S)-HETE receptor - *Achlya* steroid(antheridiol) receptor - All vertebrate steroid receptors (glucocorticoid, mineralocorticoid, androgen, progesterone, and estrogen receptors) - cytoplasmic v-erbA - Hap1 - Heat-shock transcription factor HSF-1 - p53 - PAS family members: Dioxin receptor (= AhR), Sim and HIF-1*a*

**Kinases:**	**Others:**

- Akt (= protein kinase B) - Bcr-Abl - Casein kinase II*a* catalytic subunit - Cdk4, Cdk6, Cdk9 - c-Mos - Death domain kinase RIP - eEF-2 kinase - eIF2-*a* kinases HRI, PKR, Gcn2 - ErbB2 - I*k*B kinases *a* and *b* - Insulin receptor - KSR - MEK - Mik1 - MOK, MAK, MRK - Nucleophosmin-anaplastic lymphoma kinase - PDK-1 - Pim-1 - Plk1 - pp60v-src, c-src - src related tyrosine kinases: yes, fps, fes, fgr, hck, and lck - Raf-1, B-Raf, Ste11 - Wee1, Swe1	- Actin, tubulin, myosin - Apaf-1 - apoB - Atrial natriuretic peptide receptor - Calcineurin/Cna2 (catalytic subunit) - Calmodulin - Calponin - Ctf13/Skp1 component of CBF3 - DNA polymerase *a* - eNOS, nNOS - Free *bg* subunit of G protein - G*a*0, G*a*12 - GERp95 - Macromolecular aminoacyl-tRNA synthetase complex - Macrophage scavenger receptor - Mdm2 - Nascent CFTR - Proteasome -Rreovirus protein *s*1 - Reverse transcriptase of hepatitis B virus - SV40 large T-antigen - Telomerase - Thrombin receptor (PAR-1) - Vaccina core protein 4a

**Table 2:** *Arabidopsis* Hsp90 proteins.

Nomenclature	Amino acids	Chromosome	Intracellular localization
AtHSP90.5	780	II	Chloroplast stroma
AtHSP90.6	799	III	Mitochondrial matrix space
AtHSP90.7	823	IV	Endoplasmic reticulum
AtHSP90.1	705	V	Cytoplasm
AtHSP90.4	699	V	Nucleus
AtHSP90.3	668	V	Nucleus
AtHSP90.2	728	V	Nucleus

**Table 3:** *Glycine max* Hsp90 proteins.

Nomenclature	Amino acids	Chromosome	Intracellular localization
Glyma08g44590.1	699	VIII	Nucleus and plasma membrane
Glyma18g08220.1	702	XVIII	Nucleus and plasma membrane
Glyma14g01530.1	700	XIV	Nucleus and plasma membrane
Glyma02g47210.1	702	II	Nucleus and plasma membrane
Glyma09g24410.1	699	IX	Nucleus and plasma membrane
Glyma16g29750.1	699	XVI	Nucleus and plasma membrane
Glyma08g03690.1	712	VIII	Nucleus and plasma membrane
Glyma14g40320.1	847	XIV	Endoplasmic reticulum
Glyma14g40320.2	816	XIV	Endoplasmic reticulum
Glyma14g40320.4	727	XIV	Nucleus and endoplasmic reticulum (lumen)
Glyma17g37820.1	814	XVII	Endoplasmic reticulum
Glyma02g13790.1	794	II	Chloroplast stroma and mitochondrial matrix space
Glyma02g47580.1	791	II	Mitochondrial matrix space and nucleus
Glyma01g09310.1	793	I	chloroplast stroma and mitochondrial matrix space
Glyma14g01100.1	797	XIV	Mitochondrial matrix space and nucleus

**Table 4:** *Oryza sativa* Hsp90 proteins.

Nomenclature	Amino acids	Chromosome	Intracellular localization
Os04g01740.1	703	IV	Nucleus
Os06g50300.1	812	VI	Endoplasmic reticulum
Os08g38086.3	761	VIII	Mitochondrial matrix space
Os08g39140.1	699	VIII	Nucleus
Os09g29840.1	791	IX	Mitochondrial matrix space
Os09g30412.1	699	IX	Nucleus
Os09g30418.1	830	IX	Nucleus
Os09g36420.1	1046	IX	Nucleus
Os12g32986.1	811	XII	Vacuole

**Table 5:** *Zea mays* Hsp90 proteins.

Nomenclature	Amino acids	Chromosome	Intracellular localization
Zm2G012631_T01	699	II	Nucleus and mitochondrial matrix space
Zm2G112165_T01	698	II	Nucleus and mitochondrial matrix space
Zm2G069651_T01	699	II	Nucleus and mitochondrial matrix space
Zm5G833699_T01	714	V	Nucleus and mitochondrial matrix space
Zm2G141931_T01	804	II	Endoplasmic reticulum and vacuole
Zm2G141931_T03	710	II	Endoplasmic reticulum and vacuole
Zm2G399073_T02	1001	II	Endoplasmic reticulum
Zm2G399073_T03	808	II	Endoplasmic reticulum
Zm2G141931_T04	667	II	Endoplasmic reticulum
Zm2G002220_T01	793	II	Chloroplast stroma and chloroplast thylakoid membrane
Zm5G813217_T01	758	V	Nucleus and chloroplast stroma
Zm5G813217_T03	708	V	Nucleus and mitochondrial matrix space
